# Care Coordination and Hospitalization in Older Adults With or at Risk for Cardiovascular Disease: A Randomized Clinical Trial

**DOI:** 10.1001/jamanetworkopen.2026.9110

**Published:** 2026-04-28

**Authors:** Lisa M. Kern, Joselyne E. Aucapina, Samprit Banerjee, Joanna B. Ringel, Jonathan N. Tobin, Semhar Fisseha, Helena Meiri, Jessica Han, Kelly Wu, Jamie Bialor, Madeline R. Sterling, Kurt C. Stange, Monika M. Safford, Paul N. Casale

**Affiliations:** 1 Department of Medicine, Weill Cornell Medicine, New York, New York; 2 Department of Population Health Sciences, Weill Cornell Medicine, New York, New York; 3 Clinical Directors Network, New York, New York; 4 The Rockefeller University Center for Clinical and Translational Science, New York, New York; 5 NewYork Quality Care, New York, New York; 6 Department of Family Medicine and Community Health, Case Western Reserve University, Cleveland, Ohio

## Abstract

**Importance:**

Patients with or at risk for cardiovascular disease (CVD) often see many ambulatory physicians who may not communicate with each other. Care coordinators can bridge gaps in communication among physicians, but there are too few of them for all patients who might benefit.

**Objective:**

To compare the effectiveness of 2 strategies for allocating patients with or at risk for CVD to care coordination.

**Design, Setting, and Participants:**

This randomized clinical trial performed randomization and outreach from May 15 to November 30, 2023, and completed follow-up on May 31, 2024, in an accountable care organization in New York, New York. Participants included patients who were 65 years or older, had CVD or at least 1 CVD risk factor, had highly fragmented ambulatory care the previous year (fragmentation score ≥0.85), and had been attributed by Medicare to the accountable care organization.

**Intervention:**

Usual care assigned patients to care coordinators after any hospitalization. The intervention moved the time of care coordination earlier, offering care coordination proactively (without respect to hospitalization) to those who reported problems with care coordination on a telephone survey.

**Main Outcomes and Measures:**

The primary outcome was emergency department (ED) visits or hospitalizations during follow-up. The main secondary outcome was acceptability of the intervention, followed by appropriateness, fidelity, and efficiency.

**Results:**

A total of 400 participants (202 in the intervention group and 198 in the control group) were included in the analysis. The mean (SD) age of participants was 75.8 (7.0) years; 287 (71.8%) were female. Participants had a median of 14 (IQR, 9-22) visits to 8 (IQR, 6-11) physicians during the previous year. In the intervention group, 13 participants of 49 eligible (26.5%) accepted care coordination, compared with 17 of 17 (100%) in the control group. The most common reason for declining care coordination was that participants were coordinating care themselves. There was no difference in ED visits or hospitalizations (0.25 [95% CI, 0.21-0.31] events per 100 person-days alive in the intervention group vs 0.21 [95% CI, 0.17-0.27] events per 100 person-days alive in the control group; *P* = .29).

**Conclusions and Relevance:**

In this randomized clinical trial, proactive outreach for offering care coordination in advance of hospitalization did not result in better outcomes compared with usual care offering posthospitalization coordination. Many participants declined the proactive outreach offer.

**Trial Registration:**

ClinicalTrials.gov Identifier: NCT05820295

## Key Points

**Question:**

Does adding proactive care coordination before emergencies reduce emergency department (ED) visits and hospitalizations among Medicare beneficiaries with fragmented care who have cardiovascular disease (CVD) or CVD risk factors?

**Finding:**

In this randomized clinical trial with 400 participants, most Medicare beneficiaries who were offered proactive care coordination declined, saying they were coordinating care themselves. There was no difference in the outcome of ED visits or hospitalizations.

**Meaning:**

Offering proactive care coordination by care managers in advance of hospitalization did not result in better outcomes compared with posthospitalization offers; many participants declined proactive assistance by care managers, choosing instead to do the care coordination themselves.

## Teaser

This randomized clinical trial of older adults with or at risk for cardiovascular disease (CVD) compares the effectiveness of proactive care coordination vs care coordination that occurs after hospitalization.

## Introduction

Adults with cardiovascular disease (CVD) or CVD risk factors frequently receive care from multiple physicians in different specialties (eg, primary care, cardiology, pulmonology, endocrinology, ophthalmology, nephrology, neurology).^1,2^ Fragmented care occurs when patients receive care from multiple physicians and no single physician accounts for a substantial proportion (typically a majority) of visits.^3^ Although seeing multiple physicians may be clinically appropriate, it increases the risk of gaps in communication across physicians (even in the era of electronic health records [EHRs]^4^), which can lead to excess emergency department (ED) visits and hospitalizations.^5,6,7,8,9^

Accountable care organizations (ACOs) are formal alliances of physician organizations, hospitals, and others that are incentivized to improve care coordination and reduce unnecessary ED visits and hospitalizations.^10^ ACOs often employ care coordinators (members of the medical team whose role includes facilitating communication), but there are too few care coordinators to help all who might benefit.^11^ How best to select patients for care coordination is not clear.

We sought to compare the effectiveness of 2 strategies for selecting patients with CVD or CVD risk factors and highly fragmented care for care coordinators: usual care vs usual care plus proactive outreach. Usual care typically selects patients after they have been hospitalized. While this may be appropriate, it occurs after patients have been hospitalized and does not target only those patients who perceive a need for support with care coordination.^12^ Proactive outreach involves systematically eliciting patients’ perceptions on how well their own care is being coordinated—while they are in the community, without waiting for a hospitalization to occur—and offering care management to anyone who expresses concerns. The hypothesis was that usual care plus proactive outreach would avert more ED visits and hospitalizations than usual care alone.

## Methods

### Study Design

We conducted a randomized clinical trial with a parallel design and prospective assignment, allocating participants in a 1:1 ratio. This was a type 1 hybrid effectiveness-implementation trial,^13^ with pragmatic features guided by the revised Pragmatic–Explanatory Continuum Indicator Summary (PRECIS-2) framework.^14^ The Biomedical Research Alliance of New York Institutional Review Board approved the protocol and granted waivers of informed consent and Health Insurance Portability and Accountability Act authorization because the care coordination services provided were considered standard care. The trial protocol is provided in Supplement 1. We followed the Consolidated Standards of Reporting Trials (CONSORT) reporting guideline for randomized clinical trials.

### Setting

This trial was performed in New York, New York, with NewYork Quality Care (NYQC), the Medicare Shared Savings ACO that brings together NewYork–Presbyterian Hospital, ColumbiaDoctors, and the Weill Cornell Physician Organization.^15^ NYQC contributes financial support for the care coordinators employed by its component organizations, all of whom were considered care managers, due to their advanced degrees in nursing or social work. NYQC also has data analysts who analyze Medicare claims to facilitate population health management.

### Participants

Using claims, we identified and included Medicare beneficiaries 65 years or older who (1) were attributed by Medicare to NYQC in the 12 months ending December 31, 2022; (2) did not have a diagnosis code for dementia^16^; (3) were community dwelling; (4) were not enrolled in hospice; (5) had CVD or at least 1 CVD risk factor (defined as ≥1 of the following conditions^17^: acute myocardial infarction, atrial fibrillation, diabetes, heart failure, hyperlipidemia, hypertension, ischemic heart disease, and stroke or transient ischemic attack); (6) had at least 4 ambulatory visits in the attribution year^9^; (7) did not have outlier values (>99.9th percentile) for ambulatory visits or physicians; (8) had highly fragmented ambulatory care (reversed Bice-Boxerman Index ≥0.85 [eTable 1 in Supplement 2],^18^ a cut point associated with excess hospitalizations^7^); and (9) were in the catchment area of the 3 main care management teams. We extracted demographic characteristics (age, sex, race and ethnicity, Medicare enrollment type, and additional comorbidities^17^) from claims. Race and ethnicity (categorized as Asian, Black, Hispanic, White, other, or unknown) was assessed to determine the extent of diversity in the sample, with implications for generalizability. Claims did not specify how beneficiary race or ethnicity was identified.

### Randomization

We stratified randomization by care management team; within each team, we stratified randomization by care manager (2-3 care managers per team). Randomization was conducted by 2 investigators (S.B. and J.B.R.). Allocation was not concealed, and blinding was not feasible, because the number of care managers was limited and the same care managers worked with both trial arms.

### Control and Intervention Groups

The control group received usual care, in which individuals were eligible for care management after hospitalization or physician referral. The intervention group received usual care plus proactive outreach. Proactive outreach involved as many as 5 attempts by care managers by telephone to all patients in that group, while patients were in the community, without respect to hospitalization. Care managers explained that the health system had a new initiative to improve care coordination and invited people to participate in a survey about their perceptions on how well their own care was coordinated. The survey was administered in the same call, using a previously tested instrument,^19^ with answers recorded in REDCap.^20^ If responses to any of 8 questions about care coordination suggested a problem,^19^ care managers immediately offered to help address the concern. A study that included most of the present investigators previously reported detailed survey responses,^12^ so the present report focuses on trial design, intervention, and results. Participants were not offered financial incentives, but any care management needed was provided free of charge.

Participants could accept or decline care management. If participants declined, the stated reason for declining was documented. If participants accepted, care managers followed them up for at least 30 days and documented encounters in the EHR. We asked care managers to focus on communication for the intervention group, although we allowed them to address other issues. We gave no such instructions for the usual care group.

### Timeline and Data Collection

Randomization was conducted on May 15, 2023. The trial start dates were specific to care management teams (May 17, September 11, and September 23, 2023), with variation due to delays in reliance on a single institutional review board. Following the start dates, both trial arms had a qualifying event window (eFigure 1 in Supplement 2). A qualifying event was a hospitalization (either trial arm), direct physician referral (either arm), or a survey indicating a concern about care coordination (intervention arm only). The qualifying event window lasted 3 months for the control arm or until all participants were reached for the intervention arm, which was more than 3 months for 1 team and less than 3 months for 2 teams. Patient outreach was completed November 30, 2023.

We included a 30-day wash-in period to give care managers time to provide their services. The wash-in period was staggered, beginning with the date of contact by the care manager (or last attempted contact) for each participant who had a qualifying event or the end of the qualifying event window if no qualifying event occurred.

Participants were followed up for 1 year or until May 31, 2024, whichever came first. Participants were censored if they died or were lost to follow-up (due to having too few claims with NYQC physicians). No adverse events were expected or observed.

### Outcomes

The primary outcome was the combined outcome of all-cause ED visit or all-cause hospitalization, ascertained from claims. The main secondary outcome of acceptability was the proportion of eligible participants who accepted care management. Additional secondary outcomes were ascertained by the study team and from the EHR: appropriateness (proportion of care management services requested that were in the scope of the care managers’ credentials), fidelity (proportion of participants who received care management services among those who requested them), and efficiency (total number of care management encounters per group).

### Statistical Analysis

We used descriptive statistics to show ambulatory care patterns for those participants with high fragmentation of care (who could be included) vs low fragmentation of care (who were not included). We used descriptive statistics to characterize the sample, overall and stratified by trial arm. We noted the frequency with which participants accepted or declined care management in each group and their stated reasons.

We calculated descriptive statistics for follow-up time and censoring. We calculated the absolute number of events, as well as the rate of events per 100 person-days alive, allowing more than 1 event per person. We compared trial arms using Kruskal-Wallis tests for medians, Pearson χ^2^ or Fisher exact test for percentages, and Poisson regression models for rates. We conducted a sensitivity analysis using Cox proportional hazards regression models with only 1 event (first event) per participant.

We conducted 2 post hoc subgroup analyses. First, we conducted an analysis among those in the intervention group who reported concerns about care coordination, stratified by whether they accepted or declined care management. Second, we conducted an analysis comparing those who accepted care management across the 2 trial arms.

Based on preliminary data,^6,7^ we assumed that 40% of Medicare beneficiaries with CVD or at least 1 CVD risk factor and highly fragmented care would have an ED visit or hospitalization. Setting 2-tailed α = .05 and power of 80%, we needed a sample size of 346 participants (173 per group) to detect an absolute intervention effect of ±15% difference between groups. We set a sample size of 400 to allow for attrition.

Analyses were conducted with Stata, version 18 (StataCorp LLC). Two-sided *P* ≤ .05 was considered statistically significant.

## Results

### Deriving the Sample

**Figure 1.**
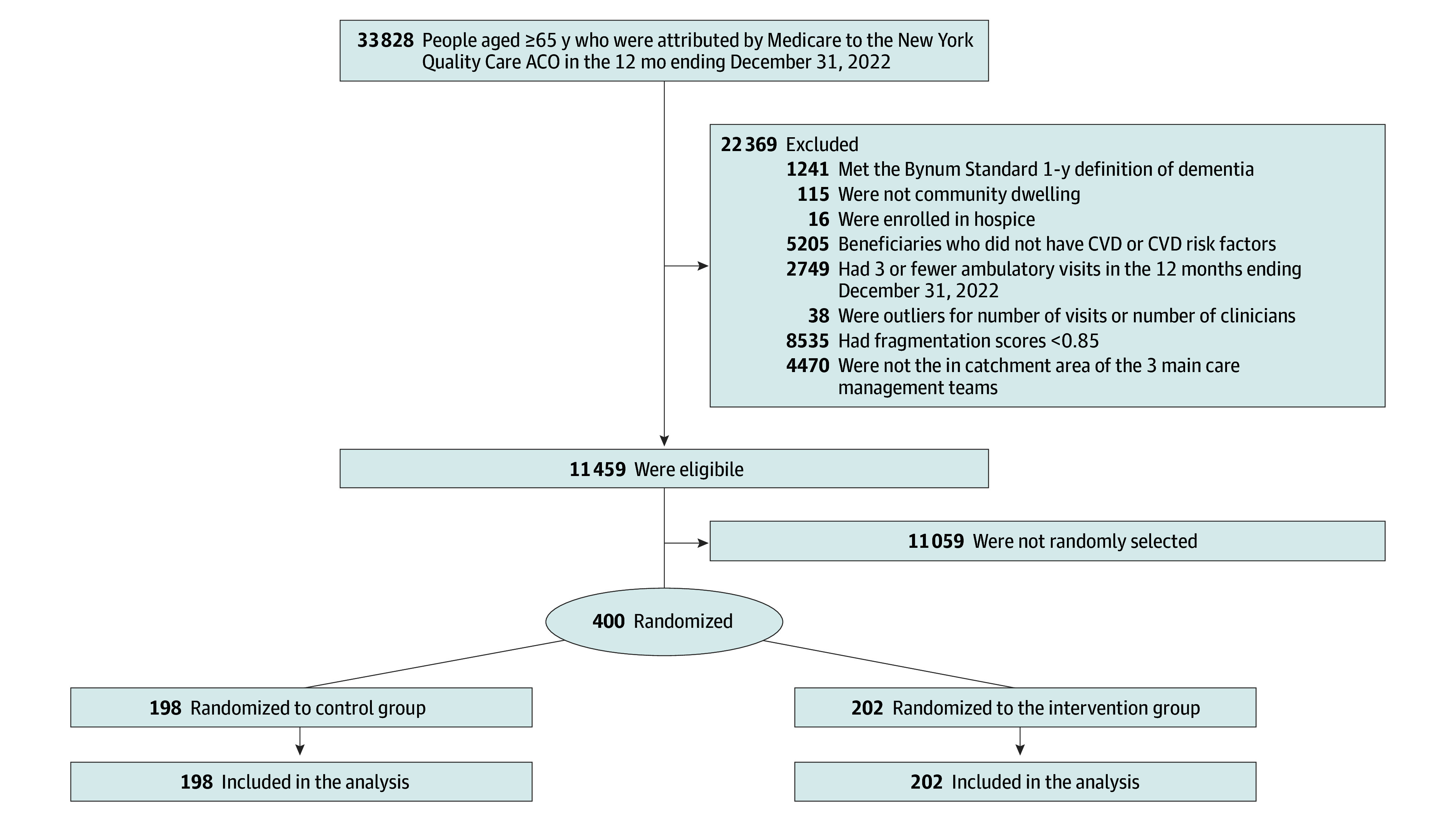
Study Flowchart The 400 randomized participants were chosen at random from the 11 459 eligible individuals. ACO indicates accountable care organization; CCW, Chronic Condition Warehouse; and CVD, cardiovascular disease.

We assessed 33 828 Medicare beneficiaries for eligibility, of whom 11 459 (33.9%) met our inclusion criteria, including having high fragmentation of care (Figure 1 and eFigure 2 in Supplement 2). eTable 2 in Supplement 2 presents the ambulatory patterns for beneficiaries with low fragmentation of care, who were not included in the study. Of the 11 459 beneficiaries who met inclusion criteria, we randomly selected 400 for the trial.

### Participant Characteristics

The 400 participants had a mean (SD) age of 75.8 (7.0) years; 287 (71.8%) were female and 113 (28.3%) were male (Table 1). In terms of race and ethnicity, 16 participants (4.0%) were Asian, 50 (12.5%) were Black, 22 (5.5%) were Hispanic, 284 (71.0%) were White, 14 (3.5%) were other race or ethnicity, and 14 (3.5%) were unknown. Two hundred sixty-three participants (65.8%) were enrolled in Medicare based on age alone. The 3 most common comorbidities were hyperlipidemia (321 [80.3%]), hypertension (297 [74.3%]), and ischemic heart disease (153 [38.3%]). Participants had a median of 14 (IQR, 9-22) visits to 8 (IQR, 6-11) different physicians, with the most frequently seen physician accounting for 24% (IQR, 19%-29%) of visits and a mean (SD) fragmentation score of 0.92 (0.04) (Table 2).

**Table 1.** Participant Characteristics

Characteristic	Participant group, No. (%)		
	Overall (N = 400)	Control (n = 198)	Intervention (n = 202)
Age, mean (SD), y	75.8 (7.0)	75.8 (7.0)	76.1 (7.1)
Sex			
Female	287 (71.8)	146 (73.7)	141 (69.8)
Male	113 (28.3)	52 (26.3)	61 (30.2)
Race and ethnicity			
Asian	16 (4.0)	9 (4.5)	7 (3.5)
Black	50 (12.5)	23 (11.6)	27 (13.4)
Hispanic	22 (5.5)	10 (5.1)	12 (5.9)
White	284 (71.0)	141 (71.2)	143 (70.8)
Other	14 (3.5)	7 (3.5)	7 (3.5)
Unknown	14 (3.5)	8 (4.0)	6 (3.0)
Enrollment type^a^			
Dual by age	136 (34.0)	67 (33.8)	69 (34.2)
Nondual by age	263 (65.8)	131 (66.2)	132 (65.4)
ESKD	1 (0.3)	0	1 (0.5)
Disabled	0	0	0
Comorbidities			
Acute myocardial infarction	3 (0.8)	3 (1.5)	0
Atrial fibrillation	47 (11.8)	28 (14.1)	19 (9.4)
CKD (any stage other than ESKD)	69 (17.3)	34 (17.2)	35 (17.3)
COPD and bronchiectasis	56 (14.0)	37 (18.7)	19 (9.4)
Colorectal cancer	13 (3.3)	4 (2.0)	9 (4.5)
Depression	87 (21.8)	44 (22.2)	43 (21.3)
Diabetes	124 (31.0)	60 (30.3)	64 (31.7)
Endometrial cancer	8 (2.0)	6 (3.0)	2 (1.0)
ESKD	4 (1.0)	3 (1.5)	1 (0.5)
Female or male breast cancer	50 (12.5)	25 (12.6)	25 (12.4)
Heart failure	59 (14.8)	30 (15.2)	29 (14.4)
Hyperlipidemia	321 (80.3)	159 (80.3)	162 (80.2)
Hypertension	297 (74.3)	143 (72.2)	154 (76.2)
Ischemic heart disease	153 (38.3)	82 (41.4)	71 (35.1)
Lung cancer	11 (2.8)	4 (2.0)	7 (3.5)
Prostate cancer	26 (6.5)	13 (6.6)	13 (6.4)
Stroke or transient ischemic attack	16 (4.0)	9 (4.5)	7 (3.5)

Abbreviations: CKD, chronic kidney disease; COPD, chronic obstructive pulmonary disease; ESKD, end-stage kidney disease; SMD, standard mean difference.

^a^ Dual indicates beneficiaries dually eligible for and enrolled in both Medicare and Medicaid.

**Table 2.** Ambulatory Patterns by Participant Group

Variable	Participant group		
	Overall (N = 400)	Control group (n = 198)	Intervention group (n = 202)
No. of ambulatory visits			
Mean (SD)	16.4 (10.3)	16.1 (9.3)	16.6 (11.2)
Median (range) [IQR]	14 (4-76) [9-22]	14 (4-50) [8-21]	13 (4-76) [9-23]
No. of ambulatory physicians			
Mean (SD)	9.2 (4.3)	9.1 (4.0)	9.4 (4.5)
Median (range) [IQR]	8 (4-25) [6-11]	8 (4-24) [6-11]	8 (4-25) [6-11]
Proportion of visits with the most frequently seen physicians			
Mean (SD)	0.24 (0.10)	0.25 (0.10)	0.24 (0.10)
Median (range) [IQR]	0.24 (0.10-0.40) [0.19-0.29]	0.25 (0.10-0.40) [0.20-0.29]	0.23 (0.10-0.40) [0.18-0.30]
Fragmentation score (reversed Bice-Boxerman Index)^a^			
Mean (SD)	0.92 (0.04)	0.92 (0.04)	0.92 (0.04)
Median (range) [IQR]	0.92 (0.85-1.00) [0.89-0.95]	0.91 (0.85-1.00) [0.89-0.94]	0.92 (0.85-1.00) [0.89-0.95]

^a^ Described in eTable 1 in Supplement 2.

### Receipt of Care Management

**Figure 2.**
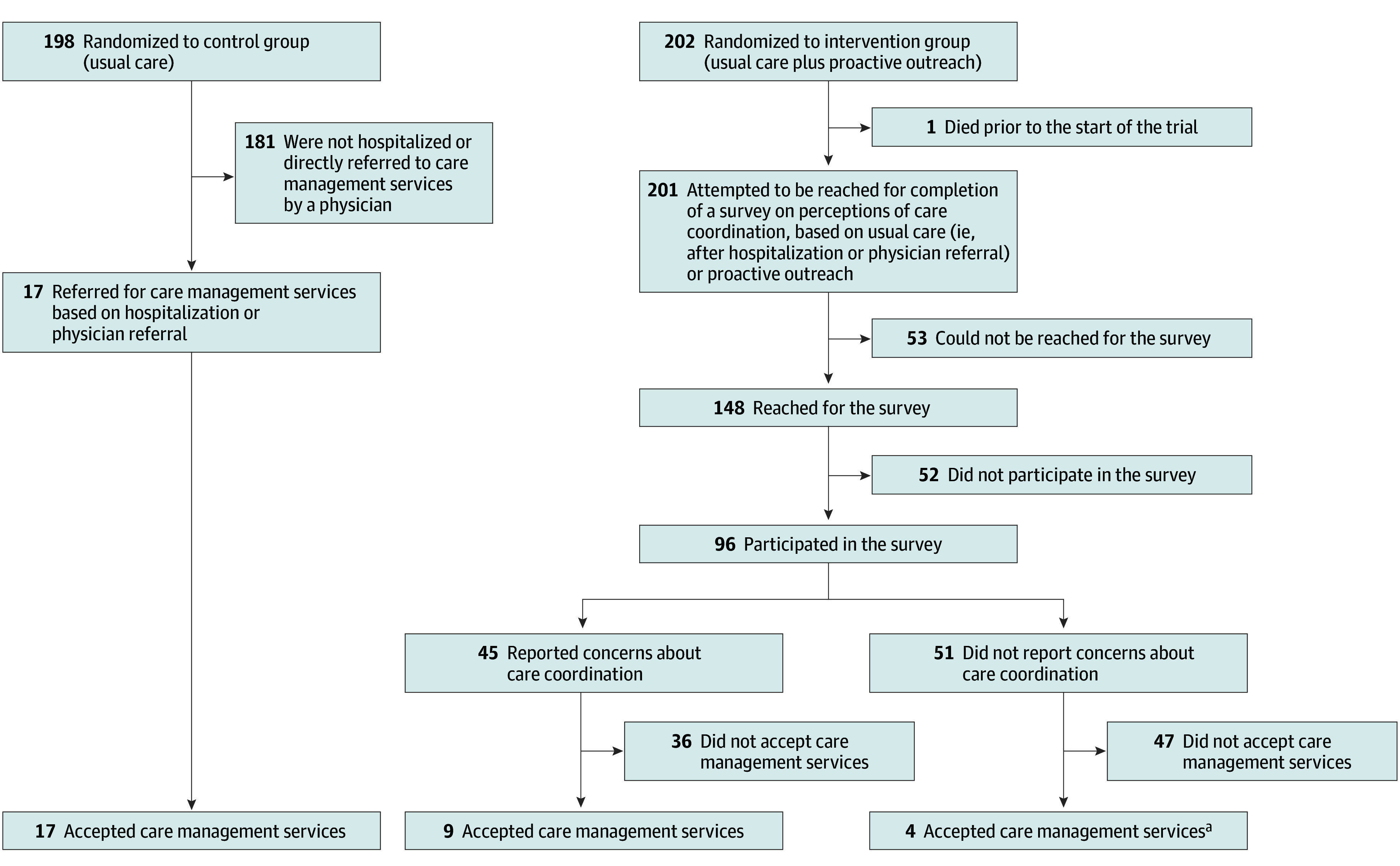
Flowchart Showing Which Participants Received Care Management No participants in the intervention group received care management without responding to the survey. ^a^Although these 4 participants did not report concerns about care coordination, they were referred to care management regardless based on hospitalization or physician referral.

Of 198 participants randomized to the control group, 17 (8.6%) were referred for care management based on hospitalization or physician referral (Figure 2 and eFigure 3 in Supplement 2). Of those, 17 participants (100%) accepted care management. Of 202 participants randomized to the intervention group, 1 died prior to the start of the trial. We attempted to reach the other 201 and succeeded for 148. Of those participants, 96 participated in the survey, 45 of 96 (46.9%) reported concerns about the coordination of their care, and 9 of 45 (20.0%) accepted care management. An additional 4 participants in the intervention group did not report concerns but were referred to care management based on hospitalization or physician referral, and all of them accepted, for a total of 13 of 49 participants (26.5%) receiving care management in the intervention group. Among those who reported concerns about the coordination of their care but declined care management, the most common reason for declining was that, while they were aware that their physicians were not communicating with each other, they were actively compensating for that by coordinating the care themselves (eTable 3 in Supplement 2). For example, one care manager’s note about why a patient declined the intervention read, “Patient is her own care coordinator since her [health system 1] specialists do not communicate with her [health system 2] primary.”

### Intention-to-Treat Analysis

Median follow-up time was 180 (IQR, 143-225) days overall (Table 3). Follow-up time was longer in the intervention than control group (median, 205 [IQR, 170-222] vs 143 [IQR, 132-260] days; *P* < .001). Of the 400 participants, 43 (10.8%) were lost to follow-up overall, including 28 (13.9%) in the intervention group and 15 (7.6%) in the control group (*P* = .04). Overall, 5 participants died during the study period, with no difference in deaths between groups.

**Table 3.** Intention-to-Treat Analysis

Variable	Participant group			*P* value^a^
	Overall	Control	Intervention	
No. of participants	400	198	202	NA
Observation (follow-up) time, d				
Median (IQR)	180 (143-225)	143 (132-260)	205 (170-222)	<.001
Range	1-350	1-349	1-350	NA
Participants who received care coordination, No. (%)	30 (7.5)	17 (8.6)	13 (6.4)	.41
Events requiring censoring, No. (%)				
Lost to follow-up in claims	43 (10.8)	15 (7.6)	28 (13.9)	.04
Deaths	5 (1.3)	1 (0.5)	4 (2.0)	.37
Outcome measures^b^				
No. of ED visits resulting in discharge	104	47	57	NA
Rate of ED events per 100 person-days alive (95% CI)	0.15 (0.12-0.18)	0.14 (0.11-0.19)	0.15 (0.12-0.20)	.64
No. of hospitalizations	62	25	37	NA
Hospitalizations per 100 person-days alive (95% CI)	0.09 (0.07-0.11)	0.07 (0.05-0.11)	0.10 (0.07-0.14)	.26
No. of ED visits or hospitalizations	166	72	94	NA
Rate of ED visits or hospitalizations per 100 person days alive (95% CI)	0.23 (0.20-0.27)	0.21 (0.17-0.27)	0.25 (0.21-0.31)	.29

Abbreviations: ED, emergency department; NA, not applicable.

^a^ *P* values to compare medians were derived from Kruskal-Wallis tests; to compare percentages, Pearson χ^2^ or Fisher exact tests; and to compare rates, Poisson regression models.

^b^ A given participant could experience multiple events.

Overall, there were 104 ED visits and 62 hospitalizations, or 166 events in total. There were 0.15 (95% CI, 0.12-0.18) ED visits per 100 person-days alive, 0.09 (95% CI, 0.07-0.11) hospitalizations per 100 person-days alive, and 0.23 (95% CI, 0.20-0.27) events per 100 person-days alive. There was no significant difference in the ED visit rate, the hospitalization rate, or the total event rate by trial arm (eg, 0.25 [95% CI, 0.21-0.31] events per 100 person-days alive in the intervention group vs 0.21 [95% CI, 0.17-0.27] events per 100 person-days alive in the control group; *P* = .29).

### Sensitivity and Subgroup Analyses

Results persisted in the time-to-event analysis, with no difference between groups (eTable 4 in Supplement 2). When we considered only those participants who were eligible for care management in the intervention group, the rate of events was lower among those who declined care management than among those who accepted (0.22 [95% CI, 0.14-0.36] events per 100 person-days alive vs 0.48 [95% CI, 0.27-0.85] events per 100 person-days alive; *P* = .04) (eTable 5 in Supplement 2). When we compared participants who accepted care management in each trial arm, there was no difference in the event rate (eTable 6 in Supplement 2).

### Secondary Outcomes

As noted previously, acceptability was lower in the intervention group (13 of 49 [26.5%]) than in the control group (17 of 17 [100%]). In terms of appropriateness, 100% of the concerns raised by the participants in both groups were in scope for the care manager’s credentials. Fidelity was 100%; all participants who requested care management received it. In terms of efficiency, care managers provided 52 encounters for the intervention group and 45 for the control group.

## Discussion

Overall, this randomized clinical trial of 400 Medicare beneficiaries with or at risk for CVD and highly fragmented ambulatory care did not find any difference in the combined outcome of ED visit or hospitalization between the trial arms. This suggests that proactive outreach did not add value to the usual approaches for selecting individuals for care management after hospitalization or physician referral.

An important finding was that acceptability of care management was considerably lower in the intervention group (26.5%) than in the control group (100%). This finding is particularly striking because the 36 eligible participants who declined care management in the intervention group declined immediately after reporting concerns about the coordination of their care. The most common reason for declining care management was that the participants were trying to address gaps in communication themselves. Thus, we found direct evidence that many patients declined care management while explicitly acknowledging that there were gaps in communication and that they were trying to address those gaps themselves.

A similar trial for people living with dementia has been recently published.^21^ Aside from that trial, which included many of the present investigators, we are not aware of any other trials that restricted inclusion to those with highly fragmented care or compared the effectiveness of 2 different methods for selecting patients for care managers. Published rates of patient engagement in care management interventions vary widely from approximately 20% to 50% of patients willing to participate.^22,23,24^ This context makes our intervention group engagement rate of 26.5% seem more typical than the 100% engagement rate we found in the control group.

Our findings indicate that some patients are intentionally trying to compensate for physicians’ failures to communicate with each other. While this may be known anecdotally, to our knowledge there is no prior empirical information indicating that patients choose to do this even when help is offered. Typically, coordination is a function of primary care,^25,26^ but the primary care system is currently overextended,^27,28,29^ which patients may sense. Guidelines have been written for patients about how to advocate for themselves with a single physician,^30^ but there is no guidance on how to communicate across multiple physicians. As recently as January 2025, the strategy of using paper binders to facilitate communication across physicians remained popular among patients; for example, an online how-to video about this strategy received more than 4.7 million views,^31^ suggesting that many patients feel obligated to coordinate care themselves.

As recently as 20 years ago, physicians wrote detailed letters to each other about each patient they were referring or had just evaluated.^32^ The responsibility for communicating information across physicians belonged to the physicians, and they were incentivized to communicate because they needed to maintain a stream of referrals.^32^ By 10 years ago, with EHRs and more consolidated health systems, customized messages for outpatient referrals had become much less common; instead, it was generally assumed that others will read the notes if they want more information.^33^ However, this approach has not solved the problem of physician-to-physician communication.^4^ While patients were always expected to contribute information about their symptoms, it was never explicitly decided by the medical community that the responsibility of communication across physicians should be shifted to patients. This may be creating an unintended burden for patients. It may also be that not all patients can navigate the health system similarly, which should be investigated in future work.

### Limitations

This study has several limitations. First, it took place in a single ACO in New York City and may not be generalizable to other settings. Second, fewer than half of patients were members of racial or ethnic minority groups or had low income levels; additional research is needed in a more diverse population. Third, follow-up time was longer and losses to follow-up were greater in the intervention group than the control group; this was an unintentional consequence of the study design, as those in the intervention group were followed up as soon as they were contacted by care management (regardless of whether they reported concerns), whereas those in the control group who were not hospitalized or referred by physicians were not followed until after the 3-month qualifying event window. This unintentional difference did not seem to affect the results, because the primary outcome was a rate, which incorporates follow-up time, and a time-to-event sensitivity analysis found results similar to those of the base case.

## Conclusions

In this randomized clinical trial, proactive outreach for care coordination did not perform better than usual care coordination. The low acceptability of the intervention and the choice by patients to coordinate their own care are important findings. Lack of effective communication across physicians is a health system problem, and we had previously assumed that it required a health system solution; however, the results of this study raise critical questions about that assumption. A national discussion is urgently needed about whose responsibility it is to communicate across physicians.

## Notes

Supplement 1.
Trial Protocol

Supplement 2. **eTable 1.** Formula for the Bice-Boxerman Index (BBI) **eFigure 1.** Timeline for the Trial, With Staggered Entry and Exit, Shown With Diagrams for the Overall Approach and Examples of Different Scenarios That Could Occur **eFigure 2.** CONSORT Flow Diagram for Pragmatic Trial on Selecting Older Adults With CVD Risk for Care Coordination **eTable 2.** Ambulatory Patterns for Community-Dwelling Medicare Beneficiaries in an Accountable Care Organization With Cardiovascular Risk Factors and ≥4 Ambulatory Visits in the Attribution Year, Stratified by Fragmentation Scores **eFigure 3.** Detailed Diagram Showing Which Participants Received Care Management **eTable 3.** Representative Quotes Indicating Reasons Participants Accepted or Declined Care Management, as Recorded by Care Managers **eTable 4.** Sensitivity Analysis Using a Time-to-Event Approach **eTable 5.** Subgroup Analyses Among Those Who Were in the Intervention Group and Eligible for Care Management Services, Stratified by Whether They Accepted or Declined Those Services **eTable 6.** Subgroup Analyses Among Those Who Received Care Management Services
eReferences

Supplement 3.
Data Sharing Statement
