# Supplementary material for: Care Coordination and Hospitalization in Older Adults With or at Risk for Cardiovascular Disease: A Randomized Clinical Trial
Source: JAMA Netw Open. 2026 Apr 28;9(4):e269110. doi: 10.1001/jamanetworkopen.2026.9110 (PMC13126219; doi:10.1001/jamanetworkopen.2026.9110)
Supplement: Supplement 1. — Trial Protocol [file jamanetwopen-e269110-s001.pdf]

CLINICAL TRIAL PROTOCOL

A. GENERAL INFORMATION

Title:	Improving How Older Adults at Risk for Cardiovascular Outcomes Are Selected for Care Coordination
Protocol/grant number:	K18 HS029255
Funding agency:	Agency for Healthcare Research and Quality (AHRQ)
Protocol version number:	2
Protocol date:	April 27, 2023
Principal Investigator:	Lisa M. Kern, MD, MPH<br>Associate Professor of Medicine<br>Weill Cornell Medicine<br>420 East 70th Street<br>New York, NY 10021<br>Phone: 646-962-5889<br>E-mail: lmk2003@med.cornell.edu

B. BACKGROUND AND OBJECTIVES

Fragmented ambulatory care (that is, care spread across multiple providers without a dominant provider) is ubiquitous in the U.S.[1] Twenty-five percent of Medicare beneficiaries ≥65 years old (approximately 9 million people) see ≥11 different ambulatory providers each year.[2-4] Having multiple providers may be clinically appropriate, but fragmented care increases the risk of gaps in communication among providers.[5] Fragmented ambulatory care has been independently associated with worse patient outcomes, especially for those at risk for cardiovascular events.[6,7] Care coordination, which involves facilitating communication among the individuals involved in a patient's care, has been used to try to overcome the problem of fragmentation.[8] However, previous care coordination interventions have had mixed results.[9] One reason for this may be that previous interventions selected patients for care coordination based on: 1) severity of illness, or 2) a transition in care (e.g., hospital discharge). While those approaches may be reasonable, they also assume that all included patients had problems with care coordination, which they may not have had. An alternate approach would select patients who experience problems with care coordination. Indeed, the PI's preliminary data show that 38% of adults ≥65 years old report problems with the coordination of their care in the past 6 months.[10] Yet, no previous intervention has leveraged these observations.

Accountable care organizations (ACOs) are highly incentivized to improve care coordination. ACOs are alliances of clinical organizations that take clinical and financial responsibility for an assigned, or attributed, patient population.[11] ACOs often have "care coordinators," who are trained professionals (often with backgrounds in nursing or social work) who facilitate communication among individuals involved in a patient's care.[12] However, ACOs typically have thousands of attributed patients and only a small number of care coordinators; for example, an ACO with 9,000 attributed patients may only have 3 care coordinators, who can care for 350 patients each or 1,050 (12%) total.[13] How to optimally allocate care coordinators is not known.

The proposed project will involve a pilot randomized controlled trial of 400 Medicare beneficiaries ≥65 years old (200 per trial arm) within an ACO, to determine the comparative effectiveness of two approaches for selecting beneficiaries for care coordination: (1) a patient-centered approach that assigns care coordinators to those beneficiaries who self-report a problem with care coordination vs. (2) usual care, which is generally after hospital discharge. The eligible population for the trial will consist of Medicare beneficiaries ≥65 years old who are attributed to NewYork Quality Care (the ACO that brings together NewYork-Presbyterian Hospital, Columbia Doctors, and the Weill Cornell Physician Organization), have highly fragmented ambulatory care (and are thus at high risk for gaps in communication), and are at risk for cardiovascular outcomes (i.e., have existing cardiovascular disease [CVD] or have ≥1 risk factor for CVD). The specific aims follow:

<u>Aim 1:</u> To determine the comparative effectiveness of two approaches for allocating care coordinators in the context of an ACO (i.e., based on self-report of problems with care coordination vs. usual care) on the combined outcome of emergency department visit or hospital admission over 12 months of follow-up

<u>Aim 2:</u> To measure implementation outcomes for the trial conducted in Aim 1 to inform future dissemination: acceptability (change in self-reported problems with care coordination after the intervention vs. before), appropriateness (frequency of different care coordination activities done), fidelity (percent of eligible individuals who receive care coordination services) and efficiency (total number of care coordinator hours spent)

This trial will be conducted in compliance with the protocol, good clinical practice, and applicable regulations.

C. TRIAL DESIGN

Primary endpoint:

The combined outcome of emergency department visit or hospital admission over 12 months of follow-up

Secondary endpoints:

(a) Acceptability (change in self-reported problems with care coordination after the intervention vs. before). (b) Appropriateness (frequency of different care coordination activities done). (c) Fidelity (percent of eligible individuals who receive care coordination services). (d) Efficiency (total number of care coordinator hours spent)

Design:

We will conduct a randomized controlled trial with a parallel design (Figure 1).

There are three main clinical entities within the ACO: Weill Cornell, Columbia, and NewYork-Presbyterian Hospital. These three entities have pre-existing care management teams, and the three entities serve different populations, based on geography within New York City and based on insurance type (such as dual eligible status). In order to remove any effect of clinical entity on the study outcome, we will conduct randomization stratified by clinical entity. We will take a random sample of people from each of the three clinical entities, such that each clinical entity will contribute one-third of the total sample.

The two study groups and the nature of the intervention are described below.

Figure 1. Overview of study design

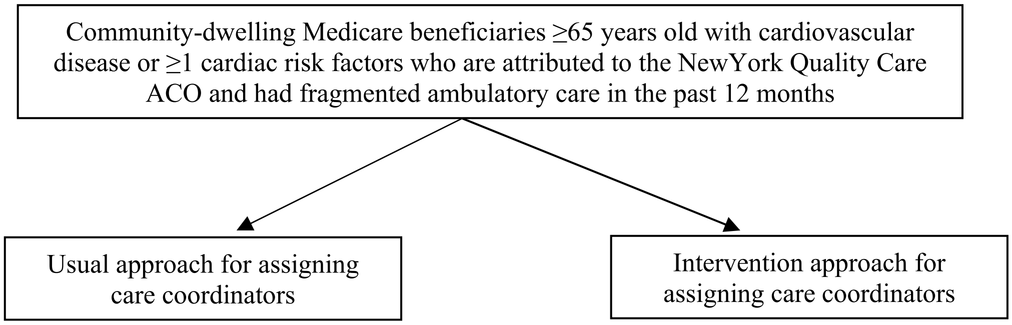

Usual care arm: The individuals who are randomized to usual care will receive care coordination services if they are identified by the ACO as eligible for them in the 3 months following randomization. Usual care consists of assigning attributed patients to care coordinators in response to: 1) a discharge from an NYQC hospital (approximately 80% of cases), 2) a discharge from a non-NYQC hospital (approximately 15% of cases, or 3) a referral from an NYQC physician asking for care coordination for a specific patient (approximately 5% of cases). An administrator at NYQC is responsible for querying daily the Epic electronic health record (EHR) to identify discharges from NYQC hospitals and for querying daily a New York City health

information exchange called NYCLIX to identify discharges from non-NYQC hospitals. The administrator assigns each eligible patient to a care coordinator, who then reaches out to the patient by phone. The care coordination process that follows is consistent with the professional standards of the American Case Management Association and the Case Management Society of America. Usual care includes describing care coordination to the patient and inviting them to engage in care coordination; approximately 70-80% of people agree to engage. Each interaction with a patient (including declining care coordination) is documented in the EHR. Usual care coordination is broad and encompasses: post-discharge medication reconciliation, inquiries about any symptoms post-discharge, a review of upcoming appointments, assistance with obtaining transportation, review of paid home care services, and any other needs that may arise, with the primary goal of preventing rehospitalization. Patients are given the care coordinator's contact information and are invited reach out with any questions or concerns. The length and frequency of interactions between the patient or proxy and the care coordinator varies depending on the patient's needs and the care coordinator's discretion. Patients are followed for a minimum of 30 days and a maximum of 1 year, which is a typical duration for care coordination.

Intervention arm: The individuals who are randomized to the intervention arm will each receive an initial call from a care coordinator in the 3 months following randomization, which we will refer to as the "screening" call. The care coordinator will explain care coordination services as they usually would and offer to administer a survey to see if the patient is eligible for care coordination. If the patient agrees, the care coordinator will administer a previously validated telephone survey on perceptions of care coordination (see Kern et al. J Gen Intern Med 2020;35:3517-24). The care coordinator will document the survey responses in customized data entry forms that we will develop in REDCap, a secure web-based data management platform. If the patient responds to the survey in a way that indicates a perceived problem with care coordination in the past 6 months (i.e., a "positive" response to greater than or equal to 1 of 8 questions, as defined previously), then we will consider this a "positive screening." The REDCap tool will notify care coordinators at the end of the survey if the survey indicates a “positive screening.” For patients and proxies whose screening calls were "negative" (not revealing a perceived problem with care coordination), the care coordinators will give them their contact information and invite them to reach out with any questions or concerns. For patients and proxies whose screening calls were "positive," care coordination will start narrowly, with the care coordinator seeking to understand the survey responses (exactly what the patient or proxy thinks is not working well and why). The care coordinator will then seek to address the issue, documenting the process in the EHR. The care coordinators will be free to engage in broader care coordination activities (e.g., general medication reconciliation, reviewing upcoming appointments, arranging transportation, etc.) at their discretion. The length and frequency of interactions between the care coordinator and patient or proxy will vary with the patient's needs and care coordinator's discretion. Patients will be followed for a minimum of 30 days and a maximum of 1 year.

Expected duration:

Participation will last up to 16 months after randomization. The events of the study are depicted below (Figure 2) and described in text that follows.

Figure 2. Timeline of events for each participant (not to scale)

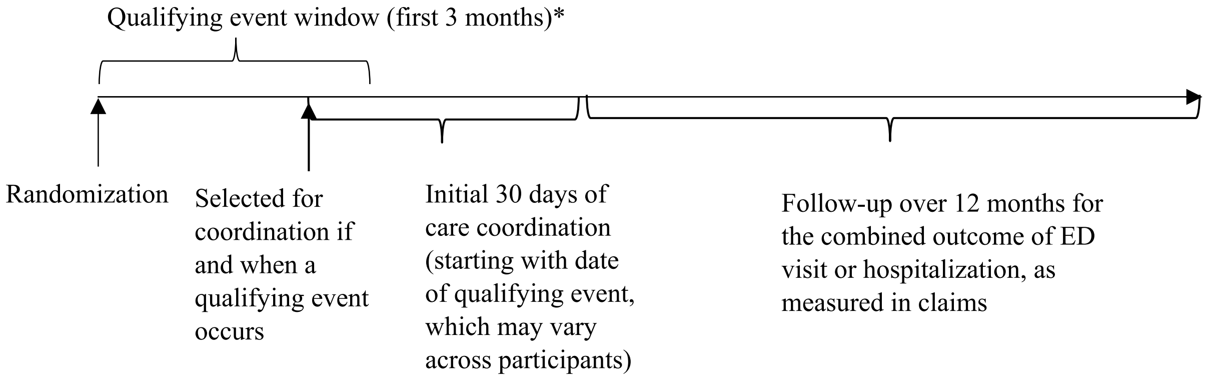

*As above, we will consider the first 3 months after randomization to be the period in which participants can have a "qualifying event." For the intervention group, the qualifying event is a survey that indicates perceived problems with care coordination. For the usual care group, the qualifying event will typically be a hospital discharge or, rarely, a physician referral. Participants who have a qualifying event will then receive attention from care coordinators, with care coordination lasting for 1 month following the qualifying event (equivalent to 2-4 months after randomization, with optional continuation of care coordination at the care coordinator's discretion). Follow-up for the combined outcome of emergency department visit or hospitalization will continue for 12 months, beginning 1 month after the start of care coordination (equivalent to starting 3-5 months after randomization and ending 15-17 months after randomization).

<u>Discontinuation:</u>

Participants may discontinue care coordination at any time.

<u>Measures to minimize bias:</u>

We will use randomization to minimize bias. Masking will not be feasible for this trial, because the care coordinators will know if a patient has been discharged from the hospital or has received a survey to assess experiences with healthcare. This seems acceptable given that it is a pragmatic clinical trial, designed to determine comparative effectiveness in the real world.

<u>Potential risks:</u>

The risks involved in this study are minimal, as the receipt of care coordination (which seeks to facilitate communication among people involved in a patient's care) is part of standard clinical practice. The only study activity that is different from usual care is the administration of a telephone survey, which takes 7 minutes to complete. This survey has previously been successfully administered to more than 7,000 adults 65 years and older nationwide, with no adverse consequences reported by the team of people who conducted the data collection for that administration. It is theoretically possible that answering the survey could increase a respondent's anxiety about the complexities of navigating healthcare, although we expect any such increase to be mild and short-lived, because we will be providing immediate resources to

address the reported problems. The other risk is a breach of confidentiality, but we can take ample measures to minimize the likelihood that this occurs, as described below.

Steps taken to minimize risk:

To minimize the likelihood that administering the survey will increase anxiety, the survey will be administered by experienced care coordinators, who have backgrounds in nursing or social work and who have years of experience with direct patient care. Care coordinators will be supervised by Dr. Paul Casale, a physician with more than 30 years of experience. To minimize the likelihood of a breach in confidentiality, we will use several methods to keep data secure. Care coordinators will enter survey data into customized forms that we will build using Research Data Electronic Capture (REDCap), a secure web-based data management platform. REDCap was developed by Vanderbilt University, supported in part by the National Institutes of Health. Weill Cornell Medicine provides its investigators with access to REDCap. All other interactions between care coordinators and study participants will be documented in the Epic electronic health record, which has its own security features. Identifiable Medicare claims will be maintained by NewYork Quality Care; any claims datasets provided to Weill Cornell Medicine for analysis will be stripped of patient identifiers prior to transfer. Access to study data will be restricted to authorized staff, the IRB, and the Office of Human Research Protections. No publications or presentations arising from this research will include personally identifiable information.

Potential benefits:

Participants in this trial may benefit directly from it. Currently, older adults generally only have access to care coordinators after they are hospitalized. The proposed intervention would provide care coordination services earlier, prior to hospitalization, if the patient identifies a need for it. Care coordination facilitates communication and has the potential to improve quality and outcomes for individuals.

There is uncertainty about how best to allocate care coordinators in the context of an ACO. ACOs may have thousands of patients but only a small number of care coordinators. To our knowledge, there have been no studies done to determine the comparative effectiveness of different approaches for assigning care coordinators to patients. This trial will generate data on the relative effectiveness of two different approaches. It will also generate data on implementation, including acceptability, appropriateness, fidelity, and efficiency. Together, these data can inform how ACOs in New York and across the country approach care coordination for their patients. This work has the potential to improve quality, efficiency, and patient outcomes for populations.

D. SELECTION AND WITHDRAWAL OF SUBJECTS

Inclusion criteria:

We will include Medicare beneficiaries greater or equal to 65 years old who: 1) are attributed to NYQC by Medicare; 2) have cardiovascular disease or greater or equal to 1 cardiovascular risk factor (defined as having one or more of the following conditions (acute myocardial infarction, atrial fibrillation, diabetes, heart failure, hyperlipidemia, hypertension, ischemic heart disease, and stroke or transient ischemic attack), 3) reside in the community, and 4) had fragmented ambulatory care in the previous 12 months. We will define fragmented care as a reversed Bice-Boxerman Index ≥0.85, a cut-point we have used previously and which typically corresponds to the top 25% of individuals.[6,14]

Exclusion criteria:

We will exclude people living with dementia (PLWD) because we are addressing care coordination needs for PLWD in a separate trial. A separate trial for that population is warranted, due to the complexity of having proxies respond to surveys and participate in care coordination. We will also exclude people who are enrolled in hospice for any reason, as those individuals are not at the same risk of going to the emergency department or having a hospital admission as the general population, given that hospice enrollment is essentially a decision not to use those services.

<u>Informed consent:</u>

We are requesting a waiver of informed consent. We believe that the study meets the 5 Common Rule criteria for a waiver, as follows.

(i) Minimal risk: The research involves no more than minimal risk to the subjects, because receipt of care coordination services is part of standard clinical care. Completing the survey is also minimal risk, as the survey does not inquire about sensitive topics. Review of data in the electronic health record and analysis of claims data are also minimal risk activities.

(ii) Practicability: The research could not practicably be carried out without the waiver. This is because, during usual care, patients are not aware of care coordinator resources unless they are assigned a care coordinator through a physician referral or after a recent discharge. If we were to try to obtain informed consent, we would need to explain care coordination, which may then raise concerns about care coordination among the participants and proxies. This would be especially problematic in the control group, which by the design of the study cannot directly request the assistance of care coordinators. This would then create problems for the feasibility and validity of the trial.

(iii) Identifiable information: The research could not practicably be carried out without the use of identifiable information. The trial relies on identifying community-dwelling Medicare beneficiaries 65 years and older who are attributed to NewYork Quality Care. We then need to reach out to those individuals who qualify for care coordination in each group, provide care coordination services, review their electronic health record data for implementation outcomes, and follow them over time for the occurrence of emergency department visits and hospital admissions as captured in claims. This study is not possible without the use of identifiers.

(iv) Rights and welfare: The waiver will not adversely affect the participants' rights. There is uncertainty about the optimal way to allocate care coordinators; this study is a comparative effectiveness trial of two approaches. Which approach is superior is currently unknown. In addition, even if a waiver is granted, eligible participants in both groups will have the option to agree to or decline care coordination services.

(v) Distribution of pertinent information after the trial: NYQC will have the opportunity to distribute the results of the trial to its attributed population. This may take the form of posters or flyers distributed in doctors' offices, a form of communication which NYQC has used previously.

If this waiver is granted, then there will be no recruitment or enrollment per se, because we will include 100% of eligible individuals. Even with a waiver of informed consent, participants in both the intervention and control groups will be able to decline care coordination services if they do not want

them. Currently, with usual care, about 70-80% of Medicare beneficiaries who are approached by the NYQC care coordinator agree to engage in care coordination. Each interaction with a patient (including declining care coordination) is documented in the EHR.

HIPAA Authorization:

We are also requesting a full waiver of HIPAA authorization for this study. We are requesting this for use of protected health information from the EHR and for use of Medicare claims for research purposes (both recruitment and outcome assessment) in compliance with the HIPAA Privacy Law. We believe that the study meets the criteria for a full HIPAA waiver, as follows:

(i) Use or disclosure involves no more than minimal risk to the privacy of individuals: We have an adequate plan in place to protect health information identifiers from improper use or disclosure. We will destroy identifiers at the earliest opportunity, absent a health or research justification or legal requirement to retain them. We also have adequate written assurances that the protected health information will not be used or disclosed to a third party, except as required by law, for authorized oversight of the research study, or for other research uses and disclosures permitted by the Privacy Rule.

(ii) Research could not practicably be conducted without the waiver: The research could not practicably be carried out without the waiver because, during usual care, patients are not aware of care coordinator resources unless they are assigned a care coordinator through a physician referral or after a recent discharge. If we were to try to obtain authorization, we would need to explain care coordination, which may then raise concerns about care coordination among the participants and proxies. This would be especially problematic in the control group, which by the design of the study cannot directly request the assistance of care coordinators. This would then create problems for the feasibility and validity of the trial.

(iii) Research could not practicably be conducted without access to and use of PHI: The research could not practicably be carried out without the use of identifiable information. The trial relies on identifying community-dwelling Medicare beneficiaries 65 years and older who are attributed to NewYork Quality Care. We then need to reach out to those individuals who qualify for care coordination in each group, provide care coordination services, review their electronic health record data for implementation outcomes, and follow them over time for the occurrence of emergency department visits and hospital admissions as captured in claims. This study is not possible without the use of identifiers.

Withdrawal:

As above, individuals can choose not to participate in care coordination at any time. Participants who no longer wish to participate would simply tell the care coordinator that they are no longer interested, and this will be documented in the EHR. We will measure the proportion of participants who discontinue care coordination services through chart review.

E. ASSESSMENT OF EFFICACY

The primary outcome (emergency department visit or hospitalization) is our main measure of effectiveness. Because this is a pragmatic trial, we aim to determine effectiveness, rather than efficacy.

F. ASSESSMENT OF SAFETY

If a care coordinator perceives that a patient or healthcare proxy experiences increased emotional stress, the care coordinator will report this to the care coordinator supervisor, who will report this to the PI. If the stress is severe and warrants additional intervention, the care coordinator will contact the patient's physician. Similarly, if the care coordinators encounter any other unexpected challenge with a patient or healthcare proxy, the care coordinator will report the situation to the care coordinator supervisor, who will report it to the PI. Of note, the research team will analyze Medicare claims monthly to monitor for the outcomes of emergency department visits and hospitalizations; these are expected events that will be tracked and reported to the PI. The PI will meet with care coordination team weekly. No interim analyses of quantitative data are planned, as the proposed trial is fairly short, with 12 months of follow-up for the combined outcome of an emergency department visit or hospital admission. Interim looks will not be sufficiently powered to be meaningful.

G. STATISTICS

Data sources:

This trial will use data from three sources: survey responses, Medicare claims (for the inclusion criterion of fragmentation and for the outcome of emergency department visit or hospital admission), and EHR chart review (for baseline demographics and for secondary outcomes).

Analysis plan:

We will track patient participation in both study groups. We will compare the characteristics of the patients in each study group, using chi-squared tests for dichotomous variables and t-tests for continuous variables, to confirm that the study groups are balanced ($p > 0.05$). We will calculate the percentage of participants in each study group with an ED visit or hospitalization (primary outcome), the numbers of ED visits and hospital admissions in each study group (sensitivity analysis), and the time-to-event in both groups (sensitivity analysis). We will use logistic regression to compare study groups for the combined outcome of any ED visit or hospital admission, adjusting for any co-variates that were imbalanced between the study groups at baseline. We will do the same with Poisson regression (or zero-inflated Poisson regression as appropriate based on the distribution of counts) for number of ED visits or hospital admissions and Cox regression to compare study groups for the time to first ED visit or hospitalization. We will conduct analyses using an intention-to-treat approach, considering all individuals as members of the group to which they were randomized. We will conduct exploratory moderator analysis with race and sex as potential moderators of treatment effect by introducing moderator x treatment interactions to our regression models.

Secondary outcomes will consist of four implementation outcomes. Acceptability will be operationalized as the change in self-reported problems with care coordination at baseline vs. 1 month after the start of care coordination services. Appropriateness will be operationalized as: a) the types of problems with care coordination uncovered in response to the survey in the intervention group, and b) the frequency of different types of care coordinator activities in each group (i.e., facilitating provider-provider communication, facilitating transportation, etc.). Fidelity will be measured by tracking how many people in the intervention group are surveyed, how many report problems with care coordination, and how many of those receive care coordination services. Implementation Cost (or Efficiency) will be measured as the total number of care coordinator hours needed in each group. Acceptability will be measured with surveys, and the pre-post comparison will be made with McNemar's test to account for the paired nature of the data. The other implementation outcomes will be measured through manual review of the electronic health record, using a standardized abstraction tool, and reported with descriptive statistics.

Sample size and power:

This trial will include Medicare beneficiaries ≥65 years old who are: 1) attributed to NewYork Quality Care by the Centers for Medicare & Medicaid Services; 2) community-dwelling; 3) who have cardiovascular disease (CVD) or ≥1 CVD risk factor (defined as one or more of acute myocardial infarction, atrial fibrillation, diabetes, heart failure, hyperlipidemia, hypertension, ischemic heart disease, and stroke or transient ischemic attack), and 4) had highly fragmented ambulatory care in the past year (as a risk factor for gaps in communication among providers). We expect that approximately 6,000 individuals will meet these inclusion criteria.

To determine how many of those 6,000 individuals we would include in the trial, we first calculated the sample size needed to determine a difference in the percentage of people in the intervention group who report a gap in care coordination at baseline vs. 1 month after receipt of care coordination services (Aim 2a). Based on our preliminary data, we assumed that 38% of people would report a gap in care coordination at baseline. Table 1 shows the sample size needed in the intervention group to find a range of effect sizes, using McNemar's test and assuming $\alpha = 0.05$, power = 80%, and correlation for paired observations of 50%. We then calculated the sample size needed to determine a significant difference in the percentage of people who experience an ED visit or hospitalization, comparing the intervention and control groups over the 12-month follow-up period (Aim 1). Using preliminary data from Dr. Kern's R01 study sample, we found that 40% of Medicare beneficiaries with CVD or ≥1 CVD risk factor and highly fragmented ambulatory care in one year had an ED visit or hospitalization the following year. Table 2 shows the sample size needed in each group to find a range of effect sizes for that outcome, using a chi-square test, $\alpha = 0.05$, and power = 80%. Based on Tables 1 and 2, we will plan to include 400 people in the trial (200 per group), which will enable us to find statistically significant and clinically meaningful differences for both Aims.

Table 1. Sample size needed in the intervention group for change in self-reported gaps in care coordination pre- vs. post intervention

Effect size	Sample size
38% ± 10%	178
38% ± 12.5%	113
38% ± 15%	79

Table 2. Sample size needed to detect difference in primary outcome between groups

Effect size	Sample size
40% ± 15%	346 (173 per group)
40% ± 20%	194 (97 per group)
40% ± 25%	124 (62 per group)

The analysis will use all randomized participants with an intention-to-treat approach. The base case analysis will use complete data, and a sensitivity analysis will use multiple imputation for missing covariates.

H. DIRECT ACCESS TO SOURCE DATA

The research team will have access to the survey data and medical records. Identifiable Medicare claims will be maintained by NewYork Quality Care; any claims datasets provided to Weill Cornell Medicine for analysis will be stripped of patient identifiers prior to transfer. Access to study data will be restricted to authorized staff, the IRB, and the Office of Human Research Protections. No publications or presentations arising from this research will include personally identifiable information.

REFERENCES

1. Elhauge E, ed. The fragmentation of U.S. health care: Oxford University Press; 2010.
2. Barnett ML, Bitton A, Souza J, Landon BE. Trends in Outpatient Care for Medicare Beneficiaries and Implications for Primary Care, 2000 to 2019. Ann Intern Med 2021;174:1658-1665.
3. Kaiser Family Foundation. Total number of Medicare beneficiaries, 2020. (Access verified on August 19, 2022, at https://www.kff.org/medicare/state-indicator/total-medicare-beneficiaries/.)
4. Pham HH, Schrag D, O'Malley AS, Wu B, Bach PB. Care patterns in Medicare and their implications for pay for performance. N Engl J Med 2007;356:1130-9.
5. O'Malley AS, Reschovsky JD. Referral and consultation communication between primary care and specialist physicians: finding common ground. Arch Intern Med 2011;171:56-65.
6. Kern LM, Rajan M, Ringel JB, et al. Healthcare Fragmentation and Incident Acute Coronary Heart Disease Events: a Cohort Study. J Gen Intern Med 2021;36:422-9.PMC7878592.
7. Kern LM, Ringel J, Rajan M, et al. Ambulatory care fragmentation and incident stroke. J Am Heart Assoc 2021;10:e019036. PMCID: PMC8200753.
8. Agency for Healthcare Research and Quality. Care coordination, 2018. (Access verified August 19, 2022, at https://www.ahrq.gov/ncepcr/care/coordination.html.)
9. Peikes D, Chen A, Schore J, Brown R. Effects of care coordination on hospitalization, quality of care, and health care expenditures among Medicare beneficiaries: 15 randomized trials. JAMA 2009;301:603-18.
10. Kern LM, Reshetnyak E, Colantonio LD, et al. Association between patients' self-reported gaps in care coordination and preventable adverse outcomes: A cross-sectional survey. J Gen Intern Med 2020;35:3517-24.PMC7728843.
11. Berwick DM. Making good on ACOs' promise--the final rule for the Medicare shared savings program. N Engl J Med 2011;365:1753-6.
12. Evans M. Demand grows for care coordinators. Modern Healthcare. March 28, 2015. (Access verified August 19, 2022, at https://www.modernhealthcare.com/article/20150328/MAGAZINE/303289980/demand-grows-for-care-coordinators.)
13. Friedman A, Howard J, Shaw EK, Cohen DJ, Shahidi L, Ferrante JM. Facilitators and Barriers to Care Coordination in Patient-centered Medical Homes (PCMHs) from Coordinators' Perspectives. J Am Board Fam Med 2016;29:90-101.PMC4809054.
14. Bice TW, Boxerman SB. A quantitative measure of continuity of care. Med Care 1977;15:347-9
