# Supplementary material for: Care Coordination and Hospitalization in Older Adults With or at Risk for Cardiovascular Disease: A Randomized Clinical Trial
Source: JAMA Netw Open. 2026 Apr 28;9(4):e269110. doi: 10.1001/jamanetworkopen.2026.9110 (PMC13126219; doi:10.1001/jamanetworkopen.2026.9110)
Supplement: Supplement 2. — eTable 1. Formula for the Bice-Boxerman Index (BBI) eFigure 1. Timeline for the Trial, With Staggered Entry and Exit, Shown With Diagrams for the Overall Approach and Examples of Different Scenarios That Could Occur eFigure 2. CONSORT Flow Diagram for Pragmatic Trial on Selecting Older Adults With CVD Risk for Care Coordination eTable 2. Ambulatory Patterns for Community-Dwelling Medicare Beneficiaries in an Accountable Care Organization With Cardiovascular Risk Factors and ≥4 Ambulatory Visits in the Attribution Year, Stratified by Fragmentation Scores eFigure 3. Detailed Diagram Showing Which Participants Received Care Management eTable 3. Representative Quotes Indicating Reasons Participants Accepted or Declined Care Management, as Recorded by Care Managers eTable 4. Sensitivity Analysis Using a Time-to-Event Approach eTable 5. Subgroup Analyses Among Those Who Were in the Intervention Group and Eligible for Care Management Services, Stratified by Whether They Accepted or Declined Those Services eTable 6. Subgroup Analyses Among Those Who Received Care Management Services eReferences [file jamanetwopen-e269110-s002.pdf]

Supplementary Online Content

Kern LM, Aucapina JE, Banerjee S, et al. Care coordination and hospitalization in older adults with or at risk for cardiovascular disease: a randomized clinical trial. *JAMA Netw Open.* 2026;9(4):e269110. doi:10.1001/jamanetworkopen.2026.9110

eTable 1. Formula for the Bice-Boxerman Index (BBI)

eFigure 1. Timeline for the Trial, With Staggered Entry and Exit, Shown With Diagrams for the Overall Approach and Examples of Different Scenarios That Could Occur

eFigure 2. CONSORT Flow Diagram for Pragmatic Trial on Selecting Older Adults With CVD Risk for Care Coordination

eTable 2. Ambulatory Patterns for Community-Dwelling Medicare Beneficiaries in an Accountable Care Organization With Cardiovascular Risk Factors and ≥4 Ambulatory Visits in the Attribution Year, Stratified by Fragmentation Scores

eFigure 3. Detailed Diagram Showing Which Participants Received Care Management

eTable 3. Representative Quotes Indicating Reasons Participants Accepted or Declined Care Management, as Recorded by Care Managers

eTable 4. Sensitivity Analysis Using a Time-to-Event Approach

eTable 5. Subgroup Analyses Among Those Who Were in the Intervention Group and Eligible for Care Management Services, Stratified by Whether They Accepted or Declined Those Services

eTable 6. Subgroup Analyses Among Those Who Received Care Management Services

eReferences

This supplementary material has been provided by the authors to give readers additional information about their work.

© 2026 Kern LM et al. *JAMA Network Open*.

eTable 1. Formula for the Bice-Boxerman Index (BBI)*

Formula[1]	Definitions of variables
$BBI = \frac{\left(\sum_{i=1}^{p} n_i^2\right) - n}{n\,(n-1)}$	where n = total number of visits in the 12-month period<br>n_i = number of visits to provider i<br>p = total number of providers

*This study used the reversed Bice-Boxerman Index, which equals 1- BBI.

© 2026 Kern LM et al. *JAMA Network Open*.

eFigure 1. Timeline for the Trial, With Staggered Entry and Exit, Shown With Diagrams for the Overall Approach and Examples of Different Scenarios That Could Occur

Panel A. Overall approach.

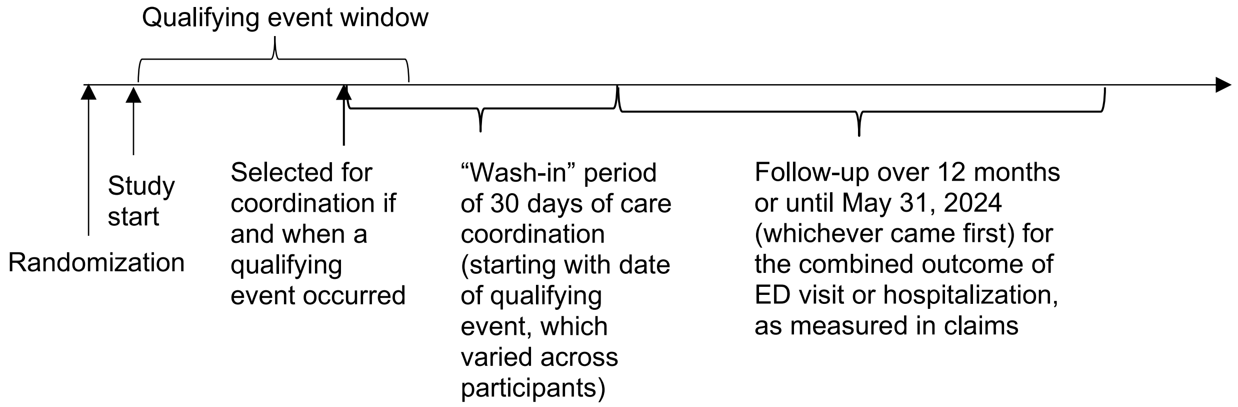

Panel B. Scenario for participants in the intervention group who completed the survey and accepted care management.

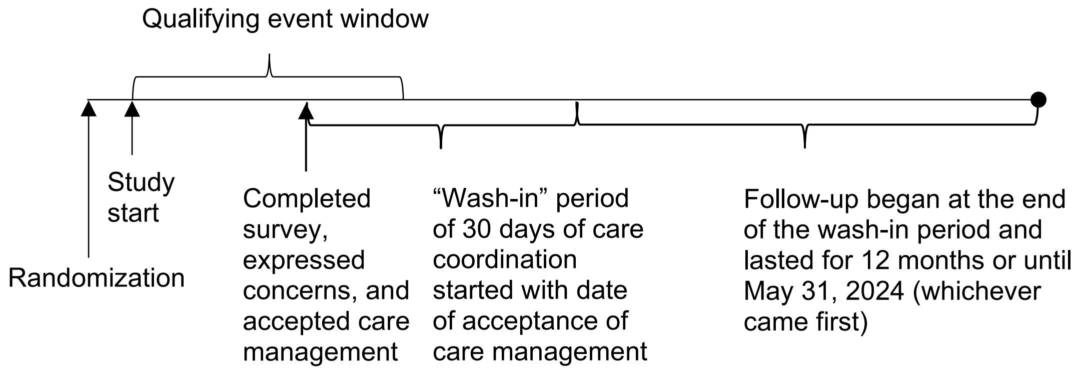

Panel C. Scenario for participants in the intervention group who were not reached for the survey, who were reached but declined to participate, who participated but did not report concerns about care coordination, or who reported concerns but declined care management.

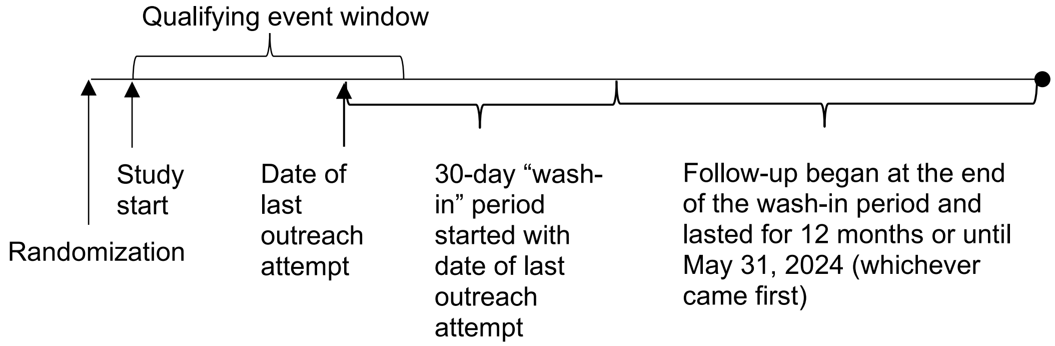

© 2026 Kern LM et al. *JAMA Network Open*.

Panel D. Scenario for participants in the control group who had a hospitalization or physician referral, whether they accepted care management or not.

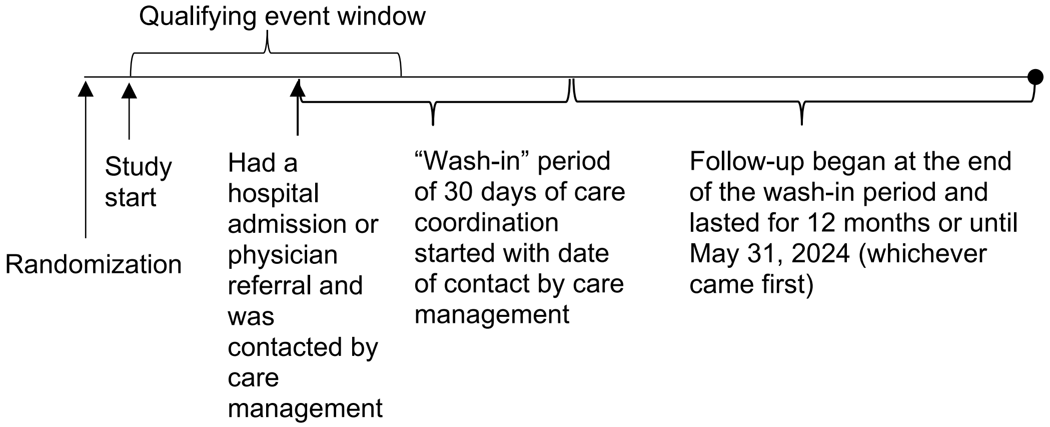

Panel E. Scenario for participants in the control group who did not have a hospitalization or physician referral.

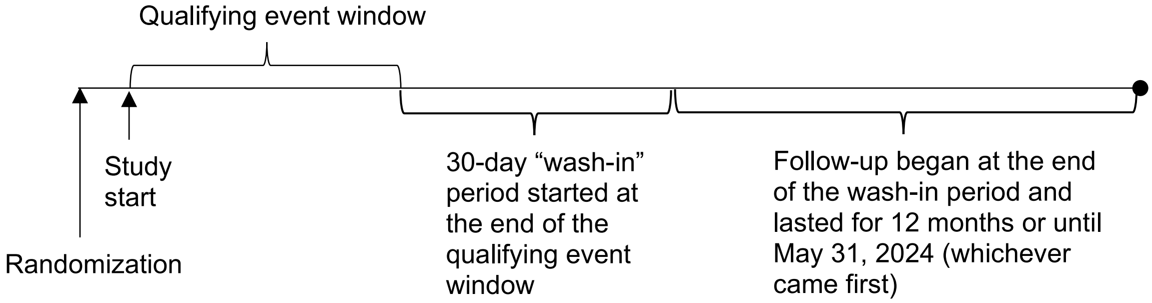

© 2026 Kern LM et al. *JAMA Network Open*.

eFigure 2. CONSORT Flow Diagram for Pragmatic Trial on Selecting Older Adults With CVD Risk for Care Coordination[2]

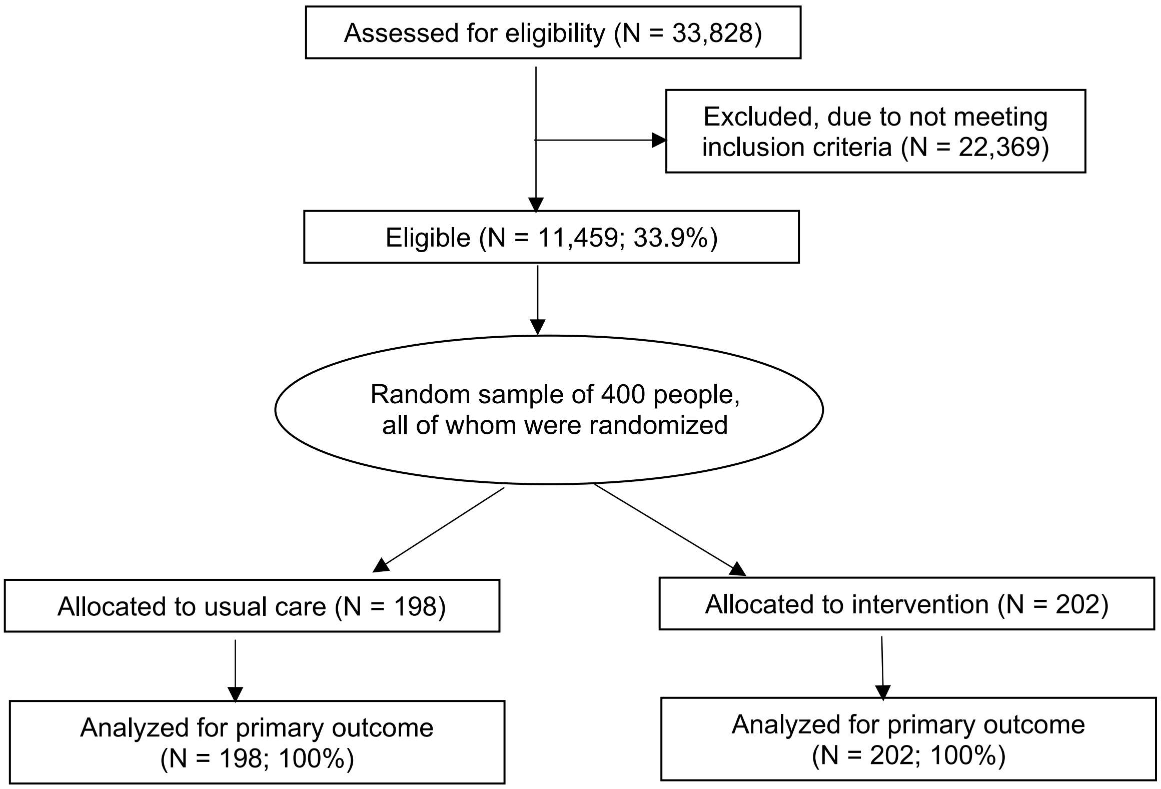

*See Figure 1 for details on how many participants were excluded for not meeting each of the inclusion criteria. See Figure 2 for details on how many people received care management in each group; because this trial was designed to determine how best to allocate scarce resources, both the people who received care management and the people who did not were included in the analysis for the primary outcome. See Table 3 for losses to follow-up; because the outcome was expressed as a rate, those lost to follow-up were included for the time that they were observed.

© 2026 Kern LM et al. *JAMA Network Open*.

eTable 2. Ambulatory Patterns for Community-Dwelling Medicare Beneficiaries in an Accountable Care Organization With Cardiovascular Risk Factors and ≥4 Ambulatory Visits in the Attribution Year, Stratified by Fragmentation Scores*

	Overall (N = 24,464)	High fragmentation (N =15,929)	Low fragmentation (N = 8,535)
Number of ambulatory visits			
Mean (sd)	14.4 (9.7)	15.4 (9.5)	12.4 (9.6)
Median (min, max)	12 (4, 82)	13 (4, 78)	10 (4, 82)
25th, 75th percentiles	8, 18	8, 19	6, 15
Number of ambulatory providers			
Mean (sd)	7.4 (3.9)	8.8 (3.9)	4.9 (2.5)
Median (min, max)	7 (1, 30)	8 (4, 30)	4 (1, 24)
25th, 75th percentiles	5, 9	6, 11	3, 6
Proportion of visits with the most frequently seen provider (%)			
Mean (sd)	33.3 (15.0)	25.1 (7.2)	48.8 (13.5)
Median (min, max)	30.3 (6.5, 100)	25.0 (6.5, 42.9)	46.7 (22.7, 100)
25th, 75th percentiles	22.2, 40.0	20.0, 30.0	40.0, 55.6
Fragmentation score (reversed Bice-Boxerman Index)			
Mean (sd)	0.85 (0.13)	0.92 (0.04)	0.73 (0.14)
Median (min, max)	0.89 (0.00, 1.00)	0.92 (0.85, 1.00)	0.78 (0.00, 0.85)
25th, 75th percentiles	0.82, 0.93	0.89, 0.94	0.69, 0.82

*The sample used in this analysis excluded those enrolled in hospice and those with outlier values for number of visits or number of providers (>99.9%ile). Fragmentation was measured with the reversed Bice-Boxerman Index, with high fragmentation defined as a score ≥0.85.

© 2026 Kern LM et al. *JAMA Network Open*.

eFigure 3. Detailed Diagram Showing Which Participants Received Care Management

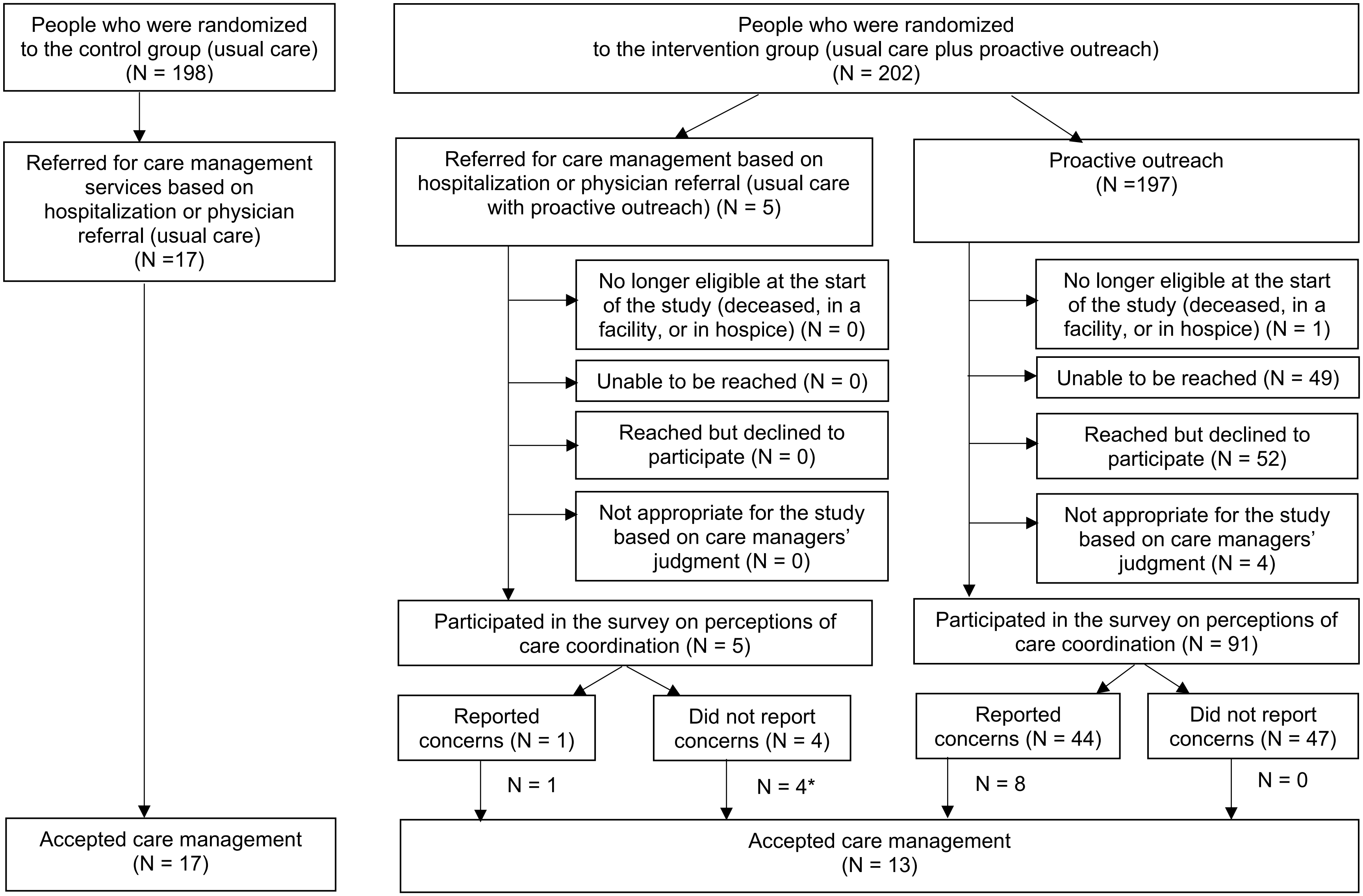

*Received care management for other reasons (e.g. post-hospitalization or physician referral).

© 2026 Kern LM et al. *JAMA Network Open*.

eTable 3. Representative Quotes Indicating Reasons Participants Accepted or Declined Care Management, as Recorded by Care Managers

Reasons for accepting care management	"Pt feels she would benefit from CM support. Sometimes has to call for results; always gets a call about results from [another health system] the next day. Pt knows doctors are busy; leaves a msg at doctor's office asking for call back but does not get call back."<br><br>"Pt has providers at [Health System 1], [Health System 2] and [Health System 3]; states not all providers are able to access records at other hospitals and pt has had duplicate tests as a result."
Reasons for declining care management	"I can manage on my own by using the patient portal or calling my provider's offices."<br><br>"Patient is her own care coordinator since her [Health system 1] specialists do not communicate with her [Health System 2] primary."<br><br>"My daughter is in charge of everything."<br><br>"I don't think a Urologist has to speak to my GI doctor, I don't think that's a thing. My GI communicates with my PCP for my colonoscopy results, I get that... I try to see my PCP when I need to. I try to keep him in the loop. It makes my life easier. When I have an appointment with my primary I try [to recall] all of my needs and relay them to the PCP."<br><br>"Pt ultimately concludes that she does her own care management."<br><br>"Providers at [Health System 1] coordinate well, however outside the system the only coordination is if she tells her doctors what's going on and she provides the documentation and test results."<br><br>"Doesn't feel he needs CM. 'Doctors are all terrific individually and we are able to coordinate when needed so we are fine.'"<br><br>"I don't want care management because it will disrupt my privacy."<br><br>"Patient can manage on her own and thinks that there are other patients that would benefit more on care management."<br><br>"The patient feels that she has good communication with her doctor and doesn't want to have someone else in the middle of her care because she feels like she can manage fine on her own."

Key: Pt = patient. CM = care manager (or care management). Msg = message. GI = gastrointestinal. PCP = primary care provider.

© 2026 Kern LM et al. *JAMA Network Open*.

eTable 4. Sensitivity analysis using a Time-to-Event Approach*

	Overall (N = 400)	Control group (N = 198)	Intervention group (N = 202)
Incident ED visits			
Number of events	69	35	34
Hazard ratio (95% confidence interval)	---	Reference	0.94 (0.58, 1.51)
Incident hospitalizations			
Number of events	43	21	22
Hazard ratio (95% confidence interval)	---	Reference	1.06 (0.58, 1.94)
Incident ED visit or hospitalization			
Number of events	91	47	44
Hazard ratio (95% confidence interval)	---	Reference	0.89 (0.59, 1.35)

*This analysis considers the first event for each participant (if any), as opposed to the main analysis which allows more than one event per person.

© 2026 Kern LM et al. *JAMA Network Open*.

eTable 5. Subgroup Analyses Among Those Who Were in the Intervention Group and Eligible for Care Management Services, Stratified by Whether They Accepted or Declined Those Services

	Total	Those who were eligible for care management in the intervention group and accepted	Those who were eligible for care management in the intervention group and declined	p-value*
N	49	13	36	
Observation time				
Median (Interquartile range) follow-up time (days)	216 (176, 229)	216 (175, 222)	215.5 (176, 230)	0.57
Range follow-up time (days)	1-350	1-232	1-350	
Events requiring censoring				
Lost to follow up in claims, N (%)	4 (8.2)	1 (7.7)	3 (8.3)	>0.99
Deaths, N (%)	1 (2.0)	0	1 (2.8)	>0.99
Outcome measures				
Number of emergency department (ED) visits (resulting in discharge, not hospitalization)	20	8	12	
Rate of ED Events per 100 person-days alive (95% CI)	0.20 (0.13, 0.31)	0.32 (0.16, 0.64)	0.16 (0.09, 0.28)	0.12
Number of hospitalizations	9	4	5	
Hospitalizations per 100 person-days alive (95% CI)	0.09 (0.05, 0.17)	0.16 (0.06, 0.43)	0.07 (0.03, 0.16)	0.18
Number of ED visits or hospitalizations	29	12	17	
Rate of ED visits or hospitalizations per 100 person days alive (95% CI)	0.29 (0.20, 0.41)	0.48 (0.27, 0.85)	0.22 (0.14, 0.36)	0.04

*P-values to compare medians were derived from the Kruskal-Wallis test, p-values to compare percentages derived were from a Pearson chi-square or Fisher’s exact test, and p-values to compare rates were derived from Poisson regression models.

© 2026 Kern LM et al. *JAMA Network Open*.

eTable 6. Subgroup Analyses Among Those Who Received Care Management Services

	Total	Those who were eligible for care management and accepted in the control group	Those who were eligible for care management and accepted in the intervention group	p-value*
N	30	17	13	
Observation time				
Median (Interquartile range) follow-up time (days)	210 (155, 218)	203 (152, 218)	216 (175, 222)	0.39
Range follow-up time (days)	1-349	139-349	1-232	
Events requiring censoring				
Lost to follow up in claims, N (%)	1 (3.3)	0 (0)	1 (7.7)	0.43
Deaths, N (%)	0	0	0	--
Outcome measures				
Number of emergency department (ED) visits (resulting in discharge, not hospitalization)	16	8	8	
Rate of ED Events per 100 person-days alive (95% CI)	0.27 (0.17, 0.44)	0.23 (0.12, 0.47)	0.32 (0.16, 0.64)	0.52
Number of hospitalizations	14	10	4	
Hospitalizations per 100 person-days alive (95% CI)	0.24 (0.14, 0.4)	0.29 (0.16, 0.54)	0.16 (0.06, 0.43)	0.31
Number of ED visits or hospitalizations	30	18	12	
Rate of ED visits or hospitalizations per 100 person days alive (95% CI)	0.51 (0.36, 0.73)	0.53 (0.33, 0.84)	0.48 (0.27, 0.85)	0.82

*P-values to compare medians were derived from the Kruskal-Wallis test, p-values to compare percentages derived were from a Pearson chi-square or Fisher's exact test, and p-values to compare rates were derived from Poisson regression models.

© 2026 Kern LM et al. *JAMA Network Open*.

eReferences

1. Bice TW, Boxerman SB. A quantitative measure of continuity of care. *Med Care* 1977;15(4):347–9.
2. Hopewell S, Chan AW, Collins GS, et al. CONSORT 2025 Statement: Updated Guideline for Reporting Randomized Trials. *JAMA* 2025;333(22):1998–2005.

© 2026 Kern LM et al. *JAMA Network Open*.
