# Supplementary material for: Care Coordination and Hospitalization in Older Adults With or at Risk for Cardiovascular Disease: A Randomized Clinical Trial
Source: JAMA Netw Open. 2026 Apr 28;9(4):e269110. doi: 10.1001/jamanetworkopen.2026.9110 (PMC13126219; doi:10.1001/jamanetworkopen.2026.9110)
Supplement: Supplement 3. — Data Sharing Statement [file jamanetwopen-e269110-s003.pdf]

Data Sharing Statement

Kern. Care Coordination and Hospitalization in Older Adults With or at Risk for Cardiovascular Disease. *JAMA Netw Open*. Published April 28, 2026.
doi:10.1001/jamanetworkopen.2026.9110

Data
Additional Information: https://clinicaltrials.gov/study/NCT05820295
Data available: No

Additional Information
Explanation for why data not available: Data abstracted from the EHR cannot be shared, as the consent to receive clinical care does not include such data sharing. Medicare claims must be used for operational purposes and for purposed related to improving care delivery, which do not include data sharing.
